# Cross-cultural Adaptation of the "Functional Activities Questionnaire - FAQ" for use in Brazil

**DOI:** 10.1590/S1980-57642011DN05040010

**Published:** 2011

**Authors:** Maria Angélica dos Santos Sanchez, Pricila Cristina Ribeiro Correa, Roberto Alves Lourenço

**Affiliations:** 1 Doutoranda, Departamento de Pós-Graduação em Ciências Médicas, Faculdade de Ciências Médicas, Universidade do Estado do Rio de Janeiro, Rio de Janeiro RJ, Brazil e Laboratório de Pesquisa em Envelhecimento Humano, GeronLab;; 2 Doutoranda em Saúde Coletiva, Instituto de Medicina Social, Universidade do Estado do Rio de Janeiro, Rio de Janeiro RJ, Brazil e Laboratório de Pesquisa em Envelhecimento Humano, GeronLab;; 3 Doutor em Saúde Coletiva, Instituto de Medicina Social, Universidade do Estado do Rio de Janeiro, Rio de Janeiro RJ, Brazil e Laboratório de Pesquisa em Envelhecimento Humano, GeronLab.

**Keywords:** cross-cultural adaptation, informant report, disability, aging

## Abstract

**Objective:**

The aim of this paper was to present the results of the first stage of cross-cultural adaptation of the Functional Activities Questionnaire (FAQ).

**Methods:**

The tool was subjected to translation and re-translation, and the test-retest reliability of a proposed version for use in Brazil was analyzed.

**Results:**

Of the 548 questionnaire respondents, a convenience sample of 68 informants was selected for retesting. Internal consistency was measured by Cronbach's alpha (0.95) while test-retest reliability was assessed using intra-class correlation (0.97). The findings have shown that FAQ is brief - averaging seven minutes to apply, easily understood and has good intra-rater test-retest reliability.

**Conclusion:**

Our results suggest this adapted version of the FAQ is a reliable and stable tool which may be useful for assessing function in Brazilian elderly. Notwithstanding, the version should be subjected to further analysis with the aim of reaching functional equivalence.

## Abstract (pt)

**Objetivo:**

Este trabalho tem como objetivo apresentar os resultados da primeira fase da adaptação transcultural do Questionário de Atividades Funcionais (FAQ).

**Métodos:**

O instrumento foi submetido à tradução e retro tradução, e a confiabilidade teste-reteste de uma versão de proposta para uso no Brasil foi analisada.

**Resultados:**

Dos 548 que responderam ao questionário, uma amostra de conveniência de 68 informantes foi selecionada para o reteste. A consistência interna e confiabilidade teste-reteste foram medidas pelo alfa de Cronbach (0,95), a correlação intra-classe (0,97). Os achados mostram que o FAQ é breve - uma média de sete minutos, de fácil compreensão e teve boa confiabilidade intra-observador no teste-reteste.

**Conclusão:**

Nossos resultados sugerem que esta versão adaptada do FAQ é um instrumento confiável e estável, e pode ser útil na avaliação de funcionalidade de idosos brasileiros; não obstante, ele deve ser submetido a uma análise mais aprofundada com o objectivo de alcançar a equivalência funcional.

## Introduction

Functional disability, although a complex term, can be defined by the presence of difficulty in performing certain activities of daily living or even the impossibility of performing them.^1^

Assessing and quantifying the functional capacity of elderly patients is fundamental for the diagnosis of clinical conditions such as dementia and mild cognitive impairment. Specifically in dementia, the diagnosis is only possible by identifying changes in the ability to perform activities of daily living (ADL's), discriminating decline, maintenance, and potential for learning tasks that have never been done before by the individual.^2^

Different approaches have been used by health professionals in order to evaluate functional decline. These include: patient self-report; observations made by members of the health care team that assists the patient; applying of formal functional evaluation scales or interview with family members. However, there are many problems which prevent adequate evaluation. Concerning patient self-report, the presence of cognitive decline may render it unreliable, and informants often disagree with the level of performance on activities of daily living claimed by patients.^2,3^

Reports from an informant who is familiar with the performance of the evaluated individual are another suggested criterion for establishing a diagnosis of loss of functional capacity.^4,5^ In Brazil, this account is often used in a non-standard way, since validated questionnaires with informants are not yet part of clinical protocols in this milieu.

Most elderly suffering from one of the many etiologies of dementia, or who have some degree of cognitive impairment, are cared for by members of their own family at home, thus placing these family members in a good position to be informants. Their testimonies are, in general, trustworthy, and they are capable of giving adequate retrospective descriptions of the abnormal symptoms presented by patients.^3,6^

The utilization of the informant-based account has important positive aspects; notwithstanding the limitations inherent to the dependence on the availability of a person who lives with the patient and knows the patient's daily routine and functional performance status.

Recent studies have sought to adapt informant report questionnaires for use in the Brazilian context; among these are the Disability Assessment for Dementia (DAD), which seeks to measure the performance of elderly with dementia on activities of daily living, not quantifying or characterizing the abnormalities, but scoring them once they are present^7,8^ and the Informant Questionnaire on Cognitive Decline in the Elderly - IQCODE-BR, which consists of an instrument which identifies functional and cognitive losses that have occurred in the last ten years.^9,10^ Among Brazilian studies in dementia involving interviews with this same purpose in mind,^11-14^ the most widely used tool for assessing functional capacity is the Functional Activities Questionnaire (FAQ) developed by Pfeffer in 1989.^15^ The preference for this instrument is likely related to its relatively short application time. However, in Brazil the informal translations of this questionnaire are used, and references always take examiners back to the original scale. Although the translations have similar characteristics to the original proposed by Pfeffer, their structure was completely changed and no studies evaluating the psychometric properties of the version in use were available.

The FAQ is a tool to be applied to an informant, and evaluates the degree of independence for the performance of ten activities of daily living. The result ranges from 0 to 30. The final score is obtained from the simple summing of the items and the higher the score the higher the level of inability to perform tasks.^15^

Since we are dealing with a widely used instrument, the adaption process should follow the soundest methodological steps, as recommended by the scientific literature. These steps include evaluation of conceptual and item equivalence; semantic, operational, measurement, and functional equivalences.^16^ This model allows for adaptation of the instruments to other cultures, but it is necessary to correctly evaluate the equivalences between the constructs, since a badly adapted instrument may not be reliable in the target population to be studied.^17^

This study aimed to perform the early stages of cross-cultural adaptation of the original FAQ, through its translation and study of its test-retest reliability.

## Methods

### Design, study site and sample

This study was conducted with a subsample of the Study of Frailty in Elderly Brazilians (FIBRA), Rio de Janeiro Section (CNPq Project / Process 555087/2006-9 and FAPERJ / Process E-26/171.469/2006). The FIBRA study, Rio de Janeiro Section, evaluated 847 elderly clients in a health care provider for at least 12 months. Participants were aged 65 years or older, of both sexes, and living in one of the neighborhoods of the northern city of Rio de Janeiro. Data collection was conducted from January 2009 to January 2010; through a single face-to-face interview with the participant. A survey of demographic data, anthropometric measures and screening tests for cognitive and functional impairment was carried out. Later, a convenience subsample was contacted by telephone for the FAQ application procedures as described below.

### Procedures for selection of study sample

Among the 847 patients included in the FIBRA-RJ study, 646 had a Mini-Mental State Exam score of 27 or less, and were contacted to answer the FAQ. Following consent of the elderly, trained recruiters made telephone contact in order to identify a knowledgeable family member or someone familiar with the performance of daily activities of the study subject. Elderly people with severe cognitive impairment whose evaluation through the FIBRA-RJ study was performed with a relative were not contacted, and instead the contact was made directly with the informant. Five hundred and forty-eight informants agreed to answer the questionnaire and 98 refused or were not located.

The test was reapplied to the first group interviewed, and this generated a convenience sample comprising 68 individuals. The interviews were redone by the same examiner between 15 and 60 days later, with the average interval being 32 days.

### Process of cross-cultural adaptation of the questionnaire

The script adaptation of this study was based on a proposal that recommends the assessment of conceptual and item equivalence; semantic; operational; measurement and functional equivalence.^16^ The model allows for the adaptation of instruments to other cultures; however, it is necessary to evaluate the equivalence of the constructs. To do so, it suggests some caution until a new version of a particular instrument appears. The adaptation of the Functional Activities Questionnaire to the Brazilian culture involved the following steps.

**Item and conceptual equivalence** - After the choice of instrument, a review committee was formed, with a group of experts composed of three professionals working in the field of geriatrics and gerontology that - based on prior knowledge of the instrument through the literature - reviewed all the items that made up the original instrument, exploring whether their different dimensions were relevant for use in the Brazilian culture.

**Semantic equivalence** - There were two independent translations to Portuguese, two independent back-translations into English and a consolidated version was prepared based on the comparison between the translations and back-translations. There were two translations and two back-translations that led to a consolidated version, after evaluation by review committee.

This group proposed changes in some of the expressions, replacing them with other phrases more suitable for the Brazilian context. For example, we can highlight the game of Bridge, which, although played in Brazil, is not popular in all social classes, therefore we opted for something more widely known in our culture. A list of the adapted words is given in Table 1.

**Table 1:** Translations and semantic adaptations of items from the "Functional Activities Questionnaire".

Original version	Translation 1	Translation 2	Test version
1. Writing checks, paying bills, balancing checkbook, keeping financial records	Preencher cheques, pagar contas, verificar o saldo no talonário de cheques, manter histórico financeiro	Preenche cheques, paga contas, faz balanço de talão de cheque, controla as necessidades financeiras	Preenche cheques, paga contas, verifica o saldo no talão de cheque, controla as necessidades financeiras
2. Making out insurance or Social Security forms, handling business affairs or papers, assembling tax records	Preencher apólices de seguro ou formulários para a previdência social, administrar negócios ou documentos de negocio, organizar documentação tributária	Faz seguro ou formulário de seguro social, lida com negócios ou documentos, reúne contas	Faz seguro (de vida, de carro, de casa), lida com negócios ou documentos, faz imposto de renda
3. Shopping alone for clothes, household necessities and groceries	Comprar sozinho roupas, itens para a casa ou fazer sozinho supermercado	Faz compras sozinho(a) roupas, utilidades domésticas e artigos de mercearia	Compra roupas, utilidades domésticas e artigos de mercearia sozinho(a)
4. Playing a game of skill such as bridge, other card games or chess or working on a hobby such as painting photography, woodwork, stamp collecting	Participar de jogo que exija habilidade tais como "bridge" ou outro jogo de cartas ou xadrez; ou praticar "hobbies" tais como pintura, fotografia, marcenaria, coleção de selos	Pratica jogos de habilidades como o buraco, algum outro jogo de cartas ou xadrez ou tem "hobbies" como pintar, fotografar, trabalhos de marcenaria ou coleciona selos	Joga baralho, xadrez, faz palavras cruzadas, trabalhos manuais ou tem algum outro passatempo
5. Heat the water, make a cup of coffee or tea, and turn off the stove	Esquentar água, fazer café ou chá e desligar o fogão	Esquenta água, faz uma xícara de café ou chá, e desliga o fogão	Esquenta água, faz café ou chá, e desliga o fogão
6. Prepare a balanced meal (e.g., meat, chicken or fish, vegetables, dessert)	Preparar uma refeição balanceada (ex. carne, galinha ou peixe, legumes, sobremesa)	Prepara uma refeição balanceada (por ex.: carne, frango ou peixe, legumes, sobremesa)	Preparar uma refeição completa (por ex.: carne, frango ou peixe, legumes, sobremesa)
7. Pay attention to, understand, and discuss the plot or theme of a one-hour television program; get something out of a book or magazine	Presta atenção, entende e discute a trama ou o tema de um programa de televisão de 1 hora, consegue entender alguma coisa de um livro ou revista	Presta atenção, entende e comenta o enredo ou tema de programas de TV, livros ou revistas	Presta atenção, entende e comenta novelas, jornais ou revistas
8. Keep track of current events, either in the neighborhood or nationally	Acompanha os acontecimentos atuais seja no bairro ou nacionalmente	Perde a conta de eventos recentes, tanto da vizinhança quanto nacionais	Acompanha os eventos atuais no bairro ou nacionalmente
9. Remember appointments, plans, household tasks, car repairs, family occasions such as birthdays or anniversaries, holidays, medications	Lembra de compromissos, planos, tarefas domesticas, consertos do carro, eventos familiares (como aniversários de nascimento ou casamento), feriados, remédios	Lembra de compromissos, planos, tarefas domésticas, conserto de automóveis, ocasiões familiares (como aniversários), férias, medicações	Lembra de compromissos, tarefas domésticas, eventos familiares (como aniversários) e medicações
10. Travel out of neighborhood; driving, walking, arranging to take or change buses and trains, planes	Sair do bairro, dirigir, andar, pegar ou trocar de ônibus, trem ou avião	Viagem para fora da vizinhança; dirigindo, caminhando, providenciando ou trocando de ônibus, trem ou avião	Sai do bairro, dirigi, anda, pega ou troca de ônibus, trem ou avião

This version, was applied to 10 informants and required several more adjustments. A new group of 10 informants was therefore interviewed. However, there was again a need for minor adjustments, as well as an instruction manual to guide the interviewer and standardize the format of questions and answers. After three tested versions, a refined version offering good comprehensibility, and whose application lasted, on average, seven minutes, was achieved. This version shall be used in the second phase of the FIBRA-RJ project in futures studies to explore the dimensional structure, and then validated in face-to-face versions and by telephone.

**Operational equivalence** - The instrument has been kept in its original format, but the application process has changed since was also administered by telephone.

**Measurement equivalence** - This has been assessed by preliminary psychometric studies of the tool's test version, comparing results with the findings of the original instrument. In this first phase the stability of tool over time was studied.

### Procedures for applying the FAQ test version

Three research assistants were trained for two weeks involving a total of 16 hours. This training consisted of reading the instruction manual and performing simulated interviews. For each interview, adjustments were made to standardized data collection.

### Procedures for evaluating test-retest reliability

To assess the stability of the questionnaire over time, reliability has been analyzed by application of the instrument on two occasions. On the first occasion, the test version of the questionnaire was applied by telephone and respondents invited for a second interview.

### Statistical procedures

The SPSS statistical package (version 11.0) was used for storage and analysis of the data. Descriptive frequencies were employed to evaluate demographic data on elderly and informants. The internal consistency of the questionnaire was measured by Cronbach's alpha coefficient, and test-retest reliability was measured by intra-class correlation coefficient (ICC) for scores on the questionnaire.

### Ethical issues

This study was approved by the Research Ethics Committee of Pedro Ernesto University Hospital of Rio de Janeiro State University. All participants read and signed the Informed Consent term.

## Results

The average duration of the questionnaire was seven minutes (SD=2.76). Among the respondents, 79.4% were women. The average age was 58 years (SD=12.9) and 75% had more than nine years of schooling. Informants were predominantly children of the patient (46.6%) or their spouse (16.2%). The descriptive statistics of the demographic characteristics and test scores of the 68 elderly are shown in Table 2.

**Table 2:** Descriptive statistics of the demographic characteristics and test scores of 68 elderly.

	Mean	SD
Age	78.90	7.64
Education (years)	8.24	5.13
MMSE Score	23.28	4.43
FAQ - Test Score	7.60	8.77
FAQ - Retest Score	8.41	9.63

MMSE: Mini-Mental State Exam; FAQ: Functional Activities Questionnaire.

With respect to the semantic evaluation, the review committee proposed some adjustments before the first pre-test with the short version, so that the questionnaire presented a format that could be more easily understood by the sample (Table 1). The internal consistency measured by Cronbach's alpha was 0.95. Interviews were repeated after 15 to 60 days, with an average of 32 days between applications (SD=12.85) and ICC was 0.97.

## Discussion

In this study, the initial stages of cross-cultural adaptation of the FAQ were described. Through a rigorous procedure that included peer review and informants, translation and back translation, a version was obtained with very similar characteristics to the original instrument, respecting both the formulation of questions, such as different response options. A high test-retest reliability was achieved.

The FAQ is a tool widely recommended for use in Brazil to assess the ability of the elderly to perform instrumental activities of daily living.^18^ Despite the absence of studies of adaptation, a translated version is available and is in general use. This version is in the form of an inventory with answer options prepared so as not to discriminate whether the capacity for a given activity was lost or consisted of a potential ability.^19^ The major limitation of these translations is the lack of a proper process of cross-cultural adaptation, which follows rigorous methodological standards.^17^

The present study is the first step towards the creation of a Brazilian version of the FAQ, properly adapted to Brazilian culture. To assess semantic equivalence, it was decided to retain the features of the original instrument as far as possible. Indeed, in the process of translation, a close proximity in the referential meaning of several items was notable. Therefore, we presumed the existence of a literal correspondence between the original instrument and its final translation.

After the first pre-test with the target population, the instrument was adjusted accordingly, so that it could correspond to the perception of the respondent and the assumed meaning of the items, assessing the difficulties understanding certain words - true fine-tuning, according to some authors.^17^ The version used was easily comprehensible, enabling a standardized and objective assessment of the individual's functioning.

With regard to operational equivalence, administration by telephone was tested, a method different from the originally proposed face to face format. However, the reliability obtained (0.95) suggests that the test version did not change the consistency of its items, obtaining good reproducibility. As there have been no reports of this nature, it was not possible to draw any comparisons. Future investigations should be conducted in order to confirm these preliminary impressions.

A relevant weakness of this study is the inexistence of other studies to allow comparisons of results and this limits conclusions about the psychometric properties of the instruments. Future investigations should be done with the objective of confirming the findings reported.

Another important aspect to note is that the literature shows the instrument as appropriate for assessing the loss of functional capacity of individuals, being able to identify potential capacity, that is, where the individual does not perform the task, but would potentially be able to do it. This is a great advantage of the instrument because, in order to evaluate functionality of the elderly it is necessary to verify not only the performance but also remaining capacity, the potential that the elderly person has to perform an activity, even if this capacity is not being used to the full.^20^ Most of the scales assessing performance in activities of daily living do not provide this feature and the assessor is obliged use a qualitative interview to reach a proper understanding of the conditions under which such disabilities are considered.

The original study suggested that the FAQ combined with a cognitive screening tool would be able to distinguish normal elderly from elderly with questionable dementia,^15^ rendering the tool potentially useful.

The present study produced a version of the FAQ for use in the Brazilian population that proved to be easy to comprehend, allowing an objective and standardized evaluation of the functional status of the individual.

In conclusion, this version of the FAQ has shown excellent reliability and stability over time, suggesting it to be a suitable tool for assessing functionality in Brazilian elderly. However, it is still necessary to complement the process of transcultural adaptation by studying its measuring equivalence. The tool is available in its entirety at www.demneuropsy.com.br.
